# Mismatch repair deficiency screening in colorectal carcinoma by a four-antibody immunohistochemical panel in Pakistani population and its correlation with histopathological parameters

**DOI:** 10.1186/s12957-017-1158-8

**Published:** 2017-06-26

**Authors:** Atif Ali Hashmi, Rabia Ali, Zubaida Fida Hussain, Naveen Faridi, Erum Yousuf Khan, Syed Muhammad Abu Bakar, Muhammad Muzzammil Edhi, Mehmood Khan

**Affiliations:** 1 Histopathology department, Liaquat National Hospital and Medical College, Karachi, Pakistan; 2 Surgery department, Rhode Island Hospital and Brown University, Providence, Rhode Island USA; 3 Medicine department, Dhaka University, Dhaka, Bangladesh

**Keywords:** Mismatch repair deficiency, Microsatellite instability, Colon, Immunohistochemistry, TILs

## Abstract

**Background:**

Microsatellite instability (MSI) operates as the second major pathway in the colorectal carcinogenesis. Although genetic testing remains the gold standard for the detection of MSI, the College of American Pathologists (CAP) recommends an initial immunohistochemical workup with a four-antibody panel (MLH1, PMS2, MSH2, and MSH6) to screen for a defective mismatch repair system. An increased trend towards young age colorectal carcinoma (CRC) has been noticed in our population over recent years; however, neither screening for MSI by immunohistochemistry (IHC)/genetic testing was done nor were its morphological features studied. We aimed to determine the frequency of mismatch repair deficiency (dMMR) by loss of IHC expression of the aforementioned enzymes in CRC patients and its correlatation with clinicopathologic parameters.

**Methods:**

This was a retrospective study conducted at Liaquat National Hospital, Karachi, between 2012 and 2015. A total of 100 cases of CRC were included in the study that underwent surgical resection. IHC stains using antibodies MLH1, PMS2, MSH2, and MSH6 were performed by DAKO EnVision method on representative tissue blocks. The results were interpreted by senior histopathologists and correlated with clinico-pathological parameters.

**Results:**

A total of 100 cases of CRC were studied that included 51 males and 49 females. Thirty-four percent (*n* = 34) of the patients showed loss of IHC staining for MMR markers. Combined loss of expression for MLH1/PMS2 were observed in 16% (*n* = 16) of the cases. Loss of MSH2/MSH6 were seen in 6% (*n* = 6) of the cases. Loss of expression for all markers were noted in 7% (*n* = 7) of the cases. There were 5% (*n* = 5) of the cases that showed isolated loss of MLH1 only. The tumors with dMMR status were significantly associated with right-sided location (*p* = 0.013), exhibited intra-tumoral lymphocytosis (*p* = 0.007), and lymphovascular invasion (*p* = 0.043). No significant association was seen with gender, age, tumor stage, grade, or other morphological features.

**Conclusion:**

The frequency of mismatch repair deficiency in CRC patients was found to be 34% in Pakistani population which warrants further genetic testing to exclude Lynch syndrome. Moreover, right-sided location and intra-tumoral lymphocyte count may be used to identify patients who may need further workup.

## Background

There are different pathways of colorectal carcinogenesis including chromosomal instability (CIN), microsatellite instability (MSI), and CpG island methylation (CIMP) with overlap between these pathways. CIN occurs in about 85% of patients with sporadic CRC and familial adenomatous polyposis (FAP) and is characterized by aneuploidy, chromosomal rearrangements, and accumulations of mutations in oncogenes and tumor suppressor genes [1]. However, MSI is most likely to be found in hereditary non-polyposis colon cancer [2] as well as in sporadic CRC.

There are different ways for repairing DNA replication errors of which one is mismatch repair (MMR) system which functions to eliminate base-base mismatches and insertion-deletion loops. To do so, at least five different MMR proteins are required including MSH2, MLH1, MSH6, PMS1, and PMS2 [3]. Any inherited or somatic mutation or epigenetic silencing of any aforementioned genes lead to MSI.

Microsatellites are tandem repeats of one to six nucleotide repeats found throughout the genome. Their instability is characterized by contractions or expansions of these sequences within DNA [4]. Mutation rates in tumor cells with dMMR are 100–1000-fold as compared with normal cells [5]. These mutations affect important growth regulatory genes, for example, TGF-B1-RII [6], TCF4, and BAX2.

A germ-line mutation in one of the MMR gene is the cause of dMMR in patients with HNPCC (Lynch syndrome) [7]. These tumors show high levels of MSI (MSI-H). Approximately 15% of sporadic colorectal cancers with no family history also exhibit MSI [8]; however, in sporadic cases, mutation of MMR genes are infrequent whereas biallelic hypermethylation of promotor of MLH1 appears to be the most important mechanism for inactivation of MMR genes [9].

Some studies have shown that individuals with MSI-H tumors (sporadic and germ line) have improved survival rates to those with microstallite stable (MSS) tumors of similar stage [10]. Also, few studies suggest that patients with MSI-H tumors are most likely to have a risk of metachronous cancers [11]. Furthermore, MSI-H tumors may be resistant to conventional chemotherapeutic agents [12]. Knowing whether the patient has Lynch syndrome is important as decisions like the extent of surgery (segmental versus total colectomy) [13] and doing other prophylactic surgeries (hysterectomy or oophorectomy), choosing appropriate therapy, and screening family member for the same mutation have to be taken.

Although genetic testing remains the gold standard for detecting MSI, the College of American Pathologists (CAP) recommends an initial IHC workup using a four-antibody panel including MLH1, MSH2, MSH6, and PMS2 which detects the presence or absence of protein products. Although MSI testing is the gold standard, due to limited resources, we preferred using immunohistochemistry as a screening modality.

In the past, survival studies have shown distinct clinico-pathological features of dMMR tumors such as poor differentiation, mucin secretion, proximal colon location, and lymphocytic infiltration are associated with favorable prognosis. Until now, no/limited data is published on the role of MMR status in Pakistani CRC patients and the relationship between MMR status and clinico-pathological features is also not certain. In this study, we aim to evaluate the role of MMR status in relation to pathological features in CRC patients.

## Method

A total of 102 primary CRC cases were included in the study. All patients underwent surgical resections between 2013 and 2015 at Liaquat National Hospital (Karachi, Pakistan). All cases were biopsy proven. Two patients had a history of pre-operative chemoradiation therapy and were excluded from the study. Moreover, no clinical information was available of patients who received chemotherapy after surgery. Finally, 100 cases were analyzed. The study was approved by the hospital ethical committee.

All slides of all cases were retrieved and were reviewed. Then, representative paraffin-fixed tissue blocks were selected that showed both tumor and adjacent non-tumor colonic epithelium.

### Immunohistochemical study

A four-antibody panel of MMR proteins including MLH1, MSH2, MSH6, and PMS2 was performed by using DAKO EnVision method on the representative paraffin-fixed tissue blocks. Four-micrometer-thick tissue sections were deparaffinized in xylene, rehydrated in alcohol, and washed in distilled water. All the antibodies were ready-to-use monoclonal antibodies provided in liquid form in a buffer containing stabilizing protein and 0.015 mol/L sodium azide (MSH6 clone, EP49; PMS2 clone, EP51; MSH2 clone, FE11; MLH1 clone, ES05). The formalin-fixed, paraffin-embedded tissue sections were pretreated with heat-induced epitope retrieval (HIER) at 97 °C for 35–40 min at high pH (50×). The slides were then incubated with the following antibodies: MLH1, MSH2, MSH6, and PMS2. Immunohistochemistry was done manually.

According to the CAP protocol for immunohistochemistry interpretation, any nuclear staining even patchy is taken as “no loss of expression” and only absolute absence of nuclear staining should be considered “loss of expression” provided internal controls are positive. Hence, carcinoma was considered dMMR when there was absence of nuclear staining for at least one protein. Adjacent normal colonic epithelium, lymphocytes, and stromal cells served as positive internal controls. Expression of proteins was then grouped into five categories: no loss of expression, loss of expression of all four proteins, combined loss of MLH1/PMS2, combined loss of MSH2/MSH6, and isolated loss of MLH1.

### Pathological analysis

Pathological records of 100 cases were reviewed. Information like patient’s age, gender, tumor laterality, lymphovascular invasion, peri-neural invasion, T stage, and N stage were obtained from the records.

### Histopathological features

One H&E slide per case was reviewed by two senior histopathologists independently. Histopathological evaluation of tumor features and host response were done using the following criteria.

#### Tumor features

##### Mucinous histology

Extracellular mucin accumulation bounded either by neoplastic epithelium or stroma. Tumors were subgrouped as mucinous histology being absent, <10%, 10–50%, and >50% of tumor area involved [14].

##### Signet ring cells

Presence of tumor cells with intracytoplasmic mucin and peripherally displaced crescent-shaped nucleus, whether present within extracellular mucin pools or infiltrating stroma.

##### Cribriform growth pattern

Neoplastic epithelial islands with sharp punched out glandular spaces. Semi-quantitative subgrouping into 10–50% and >50% was done.

##### Poor differentiation

Solid or sheet-like pattern of tumor cells in more than 70% of tumor.

##### Medullary pattern

Sheets, trabeculae, or nests of small- to medium-sized tumor cells exhibiting syncytial pattern, frequent mitosis, and abundant stromal lymphocytic infiltration.

##### Mixed growth pattern

Distinct and different growth patterns adjacent to each other in the same histological section.

##### Necrosis

Presence of dirty necrosis. Subgrouped into focal and widespread.

#### Host immune response features

##### Crohn’s-like peri-tumoral reaction

Pronounced lymphoid reaction to tumor, composed of lymphoid follicles with germinal centers at tumor edges, not associated with either mucosa or pre-existing lymph node. Two or more large lymphoid aggregates in a section were required for the presence of this feature [15].

##### Intra-tumoral lymphocytic infiltrate

The presence of small round lymphocytes within neoplastic epithelial cells. This category was subgrouped into mild to moderate (up to three intra-epithelial lymphocytes (IEL)/HPF) and marked (>3 IEL/HPF) in accordance with the CAP guidelines.

## Results

Out of total 100 CRC cases, 34% (*n* = 34) showed loss of expression of at least one MMR protein (dMMR). Seven percent (*n* = 7) of the cases showed loss of expression of all four MMR proteins; 16% (*n* = 16) showed loss of MLH1/PMS2 proteins expression; 6% (*n* = 6) showed lack of MSH2/MSH6 protein expression; and isolated loss of MLH1 was noted in 5% (*n* = 5) of the cases. No tumor showed loss of staining with MSH6, MSH2, or PMS2 protein alone.

A total of 51 males and 49 females were enrolled. No gender preponderance with MMR status was observed (*p* = 0.082). The patient ages ranged from 19 to 85 years with a median age of 53 years. Age was subgrouped into <50 and >50 years. Thirty-seven patients were younger than 50 years of which only seven had a right-sided tumor (18%; 7/37). Like gender, no significant association with age was seen.

Tumors with dMMR status were significantly associated with right-sided location (*p* = 0.013). Eighty percent of the cases (4/5) showing isolated MLH1 loss and 66% (4/6) tumors with MSH2/MSH6 loss were right sided. Seventy percent (*n* = 70) of tumors were left sided while 30% (*n* = 30) tumors were right sided. Fifty-three percent (16/30) of the right-sided tumors and 25.7% (18/70) of the left-sided tumors were dMMR.

**Fig. 1:**
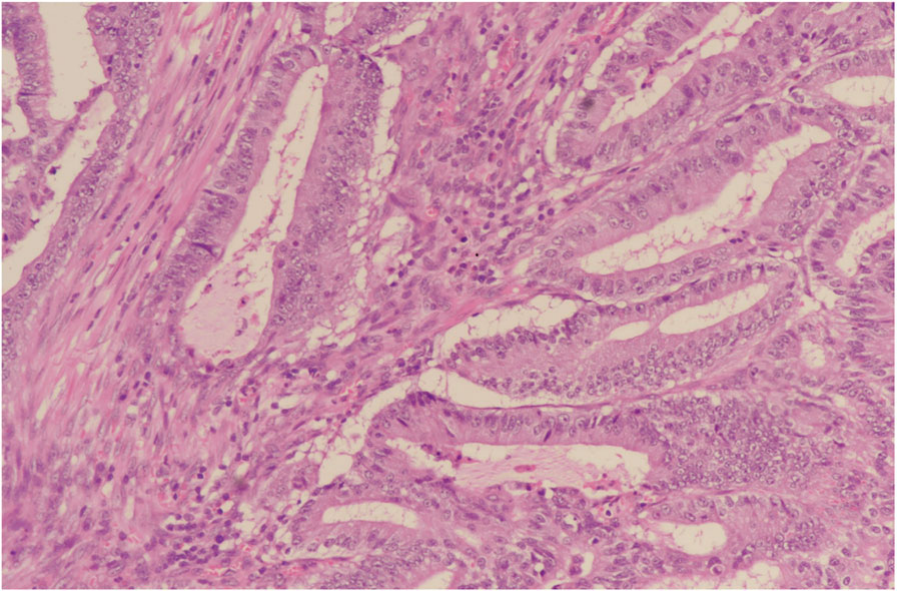
Infiltrating adenocarcinoma (H&E stain)

**Fig. 2:**
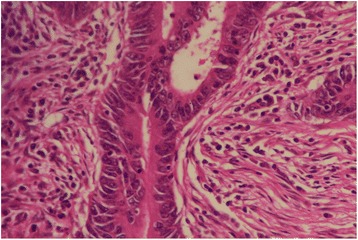
Tumor-infiltrating lymphocytes (TILS)

**Fig. 3:**
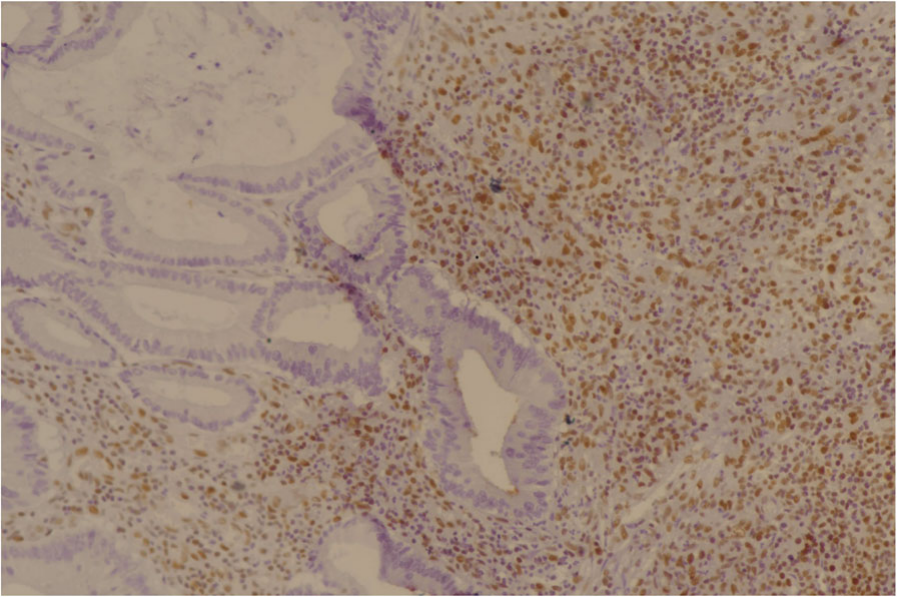
MLH1 negative with built-in control (×20)

**Fig. 4:**
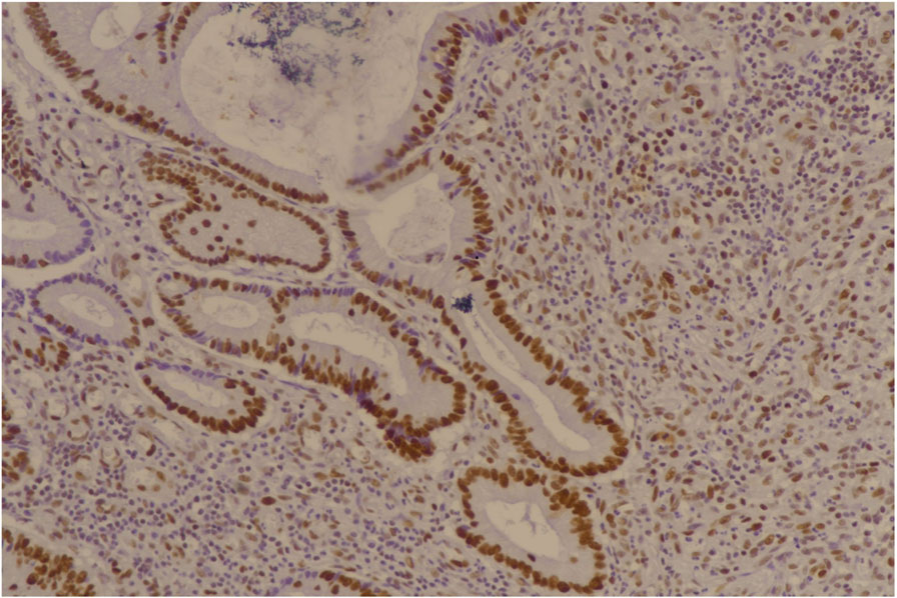
MSH6 positive (×20)

A significant association between abnormal (loss) expression of MMR proteins and tumor-infiltrating lymphocytes (TILs) was noted (*p* = 0.007). There were no tumor-infiltrating lymphocytes in 57% (*n* = 57) of tumors, while 24% (*n* = 24) exhibited mild to moderate TILs and 18% showed marked TILs. In tumors with loss of expression of all markers (*n* = 7), 72% (5/7) of the cases exhibited TILs. TILs were seen in 81% (13/16) of tumors with MLH1/PMS2 loss and 50% (3/6) of tumors with MSH2/MSH6 loss, while only 20% (1/5) of tumors with isolated MLH1 loss showed intra-tumoral lymphocytosis (Figs. 1, 2, 3, and 4).

Lymphovascular invasion was seen in 14% (1/7) of tumors with abnormal expression of all MMR proteins, 12% (2/16) of tumors with MLH1/PMS2 loss, 16% (1/6) of tumors with MSH2/MSH6 loss, and 80%(4/5) of tumors with isolated MLH1 loss. A significant association was observed (*p* = 0.043).

No significant association was seen with tumor grade or tumor type. There was only one medullary carcinoma in the study, and that showed dMMR with loss of MSH2/MSH6 (*p* = 0.004).

Similarly, no significant association was noted with T stage, N stage, signet ring or mucinous histology, poor differentiation, necrosis, or peri-tumoral Crohn’s like lymphocytic response (Table 1).

**Table 1:** Expression of MSi markers in colorectal carcinoma and its correlation with clinico-pathological features

Characteristics	No loss of expression *n* = 66	Loss of expression of all markers *n* = 7	MLH1/PMS2 loss *n* = 16	MSH2/MSH6 loss *n* = 6	Isolated MLH1 loss *n* = 5	Total *n* = 100	*p* value
Age							
<50 years	27(41%)	2(28.5%)	5(31%)	3(50%)	0(0%)	37	0.374
>50 year	39(59%)	5(71%)	11(68%)	3(50%)	5(100%)	63	
Gender							
Male	29(44%)	4(57%)	10(62%)	6(100%)	2(40%)	51	0.082
Female	37(56%)	3(43%)	6(37%)	0(0%)	3(60%)	49	
Laterality							
Right	14(21%)	2(29%)	6(37%)	4(66%)	4(80%)	30	0.013
Left	52(79%)	5(71%)	10(62%)	2(33%)	1(20%)	70	
Lymphovascular invasion							
Present	19(28.7%)	1(14%)	(12%)	1(16%)	4(80%)	27	0.043
Absent	47(71%)	6(86%)	14(88%)	5(84%)	1(20%)	73	
T stage							
T1	1(1.5%)	0(0%)	0(0%)	0(0%)	0(0%)	1	0.835
T2	3(4.5%)	1(14%)	2(12.5%)	1(16%)	0(0%)	7	
T3	55(83%)	4(57%)	13(81%)	5(84%)	4(80%)	81	
T4	7(10%)	2(29%)	1(6.2%)	0(0%)	1(20%)	11	
N stage							
N0	20(30%)	3(43%)	6(37.5%)	3(50%)	0(0%)	32	0.561
N1	21(31%)	1(14%)	6(37.5%)	1(16%)	1(20%)	30	
N2a	11(16%)	1(14%)	4(25%)	1(16%)	2(40%)	19	
N2b	14(21%)	2(29%)	0(0%)	1(16%)	2(40%)	19	
Tumor grade							
I	1(1.5%)	1(14%)	1(6.2%)	0(0%)	0(0%)	3	0.487
II	50(76%)	3(43%)	14(87%)	4(66%)	3(60%)	74	
III	15(23%)	3(43%)	1(6.2%)	2(33%)	2(40%)	23	
Tumor type							
NOS	56(84.8%)	4(57%)	15(94%)	3(50%)	2(40%)	80	0.177
Mucinous	7(12.5%)	2(28.5%)	1(6%)	2(33%)	2(40%)	14	
Medullary	0(0%)	0(0%)	0(0%)	1(16%)	0(0%)	1	
Signet ring	3(4.5%)	1(14%)	0(0%)	0(0%)	1(20%)	5	

Personal and family history suggestive of inherited cancer susceptibility was revealed in six cases, most of which were associated with MSH2/MSH6 loss as shown in Table 2 (*p* value <0.001).

**Table 2:** Expression of MSi markers in colorectal carcinoma and its correlation with histological parameters

Characteristics	No loss of expression *n* = 66	Loss of expression of all markers *n* = 7	MLH1/PMS2 loss *n* = 16	MSH2/MSH6 loss *n* = 6	Isolated MLH1 loss *n* = 5	Total *n* = 100	*p* value
Mucinous histology							
<10%	6(9%)	3(43%)	2(12.5%)	0	1(20%)	12	0.334
10–50%	4(6%)	1(14%)	1(6.25%)	1(17%)	0	7	
>50%	7(11%)	1(14%)	2(12.5%)	2(33%)	1(20%)	13	
Absent	49(74%)	2(29%)	11(69%)	3(50%)	3(60%)	68	
Signet ring differentiation							
Present	10(15%)	1(14%)	4(25%)	0	1(20%)	16	0.696
Absent	56(85%)	6(86%)	12(75%)	6(100%)	4(80%)	84	
Poor differentiation							
Present	4(6%)	1(14%)	1(6.25%)	2(33%)	1(20%)	9	0.188
Absent	62(94%)	6(86%)	15(94%)	4(67%)	4(80%)	91	
Medullary differentiation							
Present	0	0	0	1(17%)	0	1	0.004
Absent	65(98%)	7(100%)	16(100%)	5(83%)	5(100%)	99	
Necrosis							
Focal	47(71%)	4(57%)	10(63%)	5(83%)	4(80%)	70	0.198
Widespread	17(26%)	1(14%)	5(31%)	0	1(20%)	24	
Absent	2(3%)	2(29%)	1(6.25%)	1(17%)	0	6	
Tumor-infiltrating lymphocytes							
None	45(68%)	2(28%)	3(19%)	3(50%)	4(80%)	57	0.007
Mild to moderate	15(22.7%)	3(43%)	6(81%)	1(50%)	0	25	
Marked	6(9%)	2(28%)	7(81%)	2(50%)	1(20%)	18	
Peri-tumoral lymphocytic response							
None	45(68%)	5(71%)	11(69%)	2(33%)	4(80%)	67	0.454
Mild to moderate	13(20%)	1(14%)	2(13%)	1(17%)	1(20%)	18	
Marked	8(12%)	1(14%)	3(19%)	3(50%)	0	15	

## Discussion

The current study determined the frequency of dMMR status in CRC in Pakistani population which turned out to be 34%. Furthermore, tumors with dMMR status had distinct clinico-pathological features.

It is now known that a significant proportion of sporadic CRC arise through a MSI pathway characterized by defect in MMR genes. Our study demonstrates that dMMR status in CRC may be characteristic of Lynch syndrome and of patients with favorable prognosis and better survival after adjuvant chemotherapy [16]. This group of patients may be recognized on the basis of histopathology together with IHC. While PCR amplification of microsatellite repeats remain the gold standard for recognition of MSI phenotype, this approach is not feasible in routine pathology lab. Since MSI-H CRC share some morphological features (young patient age, right-sided location, mucinous and signet ring histology, intra-tumoral lymphocytosis), careful observation of tumor histology can help identify these tumors [17]. A study used parameters of right-sided location and TILs (with a positive predictive value of 57% and a negative predictive value of 95%) to identify MSI tumors. However, morphology alone would miss up to 40% of MSI-H tumors. Hence, the study states that IHC detection of protein products is a highly specific approach to pick MSI-H tumors [18].

In our study, tumors with dMMR status accounted for 34% of the total cases. Concurrent loss of MLH1/PMS2 was the most common pattern of abnormal protein expression followed by concurrent MSH2/MSH6 loss. This was similar to another study [19]. The frequency of loss of expression was found to be quite variable in different studies. It was 21% in Singapore population [20], 6.9% in Chinese population [21], and approximately 15% in western studies [22].

MSI-H cancers often evoke a host response resulting in migration of activated T cells into neoplastic epithelium^xviii^. The immune system recognizes neoplasia poorly, but in MSI-H cancers with TILs, the mechanisms of T cell cytotoxicity are activated [23]. The T cells are CD8+, TCR+ cells. Whether improved prognosis of MSI-H colonic cancers is related to upregulated immune system that prevents emergence of metastatic deposit is not known. Tumor-infiltrating lymphocytes have been independently associated with improved survival after curative surgery [24]. In our study, a significant association of dMMR was seen with tumor-infiltrating lymphocytes (*p* = 0.007). Thomas in his study concluded that quantification of TILs may provide a simple, single criterion for choosing CRC patients as candidates for MSI testing. According to his study, consideration of TILs could reduce the number of CRC referred for MSI testing by one half, yet 93% of MSI-H cancers would be identified [25]. According to Greenson et al., TILs can correctly classify tumors as MSI-H with approximately 85% probability [26].

We, like Alexander et al. [27], found that presence of peri-tumoral Crohn’s-like lymphocytic response (PTL) was an insensitive marker for MSI-H tumors. Some studies have identified this feature as an independent prognostic variable^xvi^. The frequency of detecting PTL among CRC cases in our study might be limited due to examination of only one slide per case.

In our study, 30% (30/100) of tumors were located on the right side (from cecum to splenic flexure), out of which 53% (16/30) were dMMR. While of the 70 left-sided tumors, only 25.7% (18/70) showed loss of expression of MMR proteins. Hence, a strong association of dMMR was seen with right-sided location (*p* = 0.013). A study suggests that MSI screening should be done for right-sided colon cancers in patients younger than 60 years [28].

MSI-H CRC has characteristic profile typically forming right-sided, lymphocyte rich tumors that are often mucinous [29]. Many studies have shown significant association between dMMR status and mucinous histology [30]. MSI-H mucinous tumors have better prognosis than MSS mucinous tumors. In our study, no significant association was found with mucinous histology.

In the current study, cases of both sporadic and hereditary CRC were included. Approximately 10–15% of sporadic colon cancers are MSI-H; this is due to somatic hMLH1 promotor hypermethylation resulting in epigenetic silencing and absent protein expression [31]. In our study, five cases showed isolated loss of MLH1. All patients were above 50 years of age at diagnosis. Two cases exhibited mucinous histology, and one was poorly differentiated. Eighty percent of the cases (4/5) were right sided. Such cases warrant testing for BRAF V600E mutation and MLH1 promotor hypermethylation testing prior to MSI testing.

In our study, we observed that out of 34 cases that showed any loss of expression, 61.7% (21/34) of the cases showed MLH1 loss with or without concurrent PMS2 loss. Of these cases, only one (4%; 1/21) case had a family history of colonic or endometrial cancer. We also observed that MLH1/PMS2 loss was the most frequent pattern while MSH2/MSH6 loss was less frequent and more significantly associated with inherited cancer susceptibility.

According to our results, tumors with dMMR status are more often located on the right side and are lymphocyte rich. Other established features of MSI like younger age, female gender, mucinous and signet ring morphology, or poor differentiation showed no significant correlation with dMMR status.

## Conclusions

The prevalence of abnormal expression of MMR proteins in Pakistani population was quite high as compared to international literature. In all cases of CRC, all histological parameters must be evaluated. If a tumor is right sided and exhibits tumor-infiltrating lymphocytes, then tissue should be subjected to immunohistochemistry using a four-antibody panel. And if required, MSI testing should be done if Lynch syndrome is expected.

## Abbreviations

CAP: College of American Pathologists
dMMR: Mismatch repair deficiency
MSI: Microsatellite instability
